# Quantum size effects in ultra-thin YBa_2_Cu_3_O_7 − x_ films

**DOI:** 10.1038/s41598-024-73207-z

**Published:** 2024-09-27

**Authors:** M. Lyatti, I. Gundareva, T. Röper, Z. Popović, A. R. Jalil, D. Grützmacher, T. Schäpers

**Affiliations:** 1 Peter Grünberg Institut (PGI-9), Forschungszentrum Jülich, 52425 Jülich, Germany; 2 JARA-Fundamentals of Future Information Technology, Jülich-Aachen Research Alliance , Forschungszentrum Jülich and RWTH Aachen University, Jülich, Germany; 3 Faculty of Physics, University of Belgrade, Studentski trg. 12, Belgrade, 11001 Serbia; 4 Peter Grünberg Institut (PGI-10), Forschungszentrum Jülich, 52425 Jülich, Germany

**Keywords:** Superconducting properties and materials, Single photons and quantum effects, Surfaces, interfaces and thin films, Superconducting devices

## Abstract

The d-wave symmetry of the order parameter with zero energy gap in nodal directions stands in the way of using high-temperature superconductors for quantum applications. We investigate the symmetry of the order parameter in ultra-thin YBa_2_Cu_3_O_7 − x_ (YBCO) films by measuring the electrical transport properties of nanowires aligned at different angles relative to the main crystallographic axes. The anisotropy of the nanowire critical current in the nodal and antinodal directions reduces with the decrease in the film thickness. The Andreev reflection spectroscopy of the nanoconstrictions shows the presence of a thickness-dependent energy gap that does not exist in bulk YBCO. We find that the thickness-dependent energy gap appears due to the quantum size effects in ultra-thin YBCO films that open the energy gap along the entire Fermi surface. The fully gapped state of the ultra-thin YBCO films makes them a promising platform for quantum applications, including quantum computing and quantum communications.

## Introduction

**Fig. 1:**
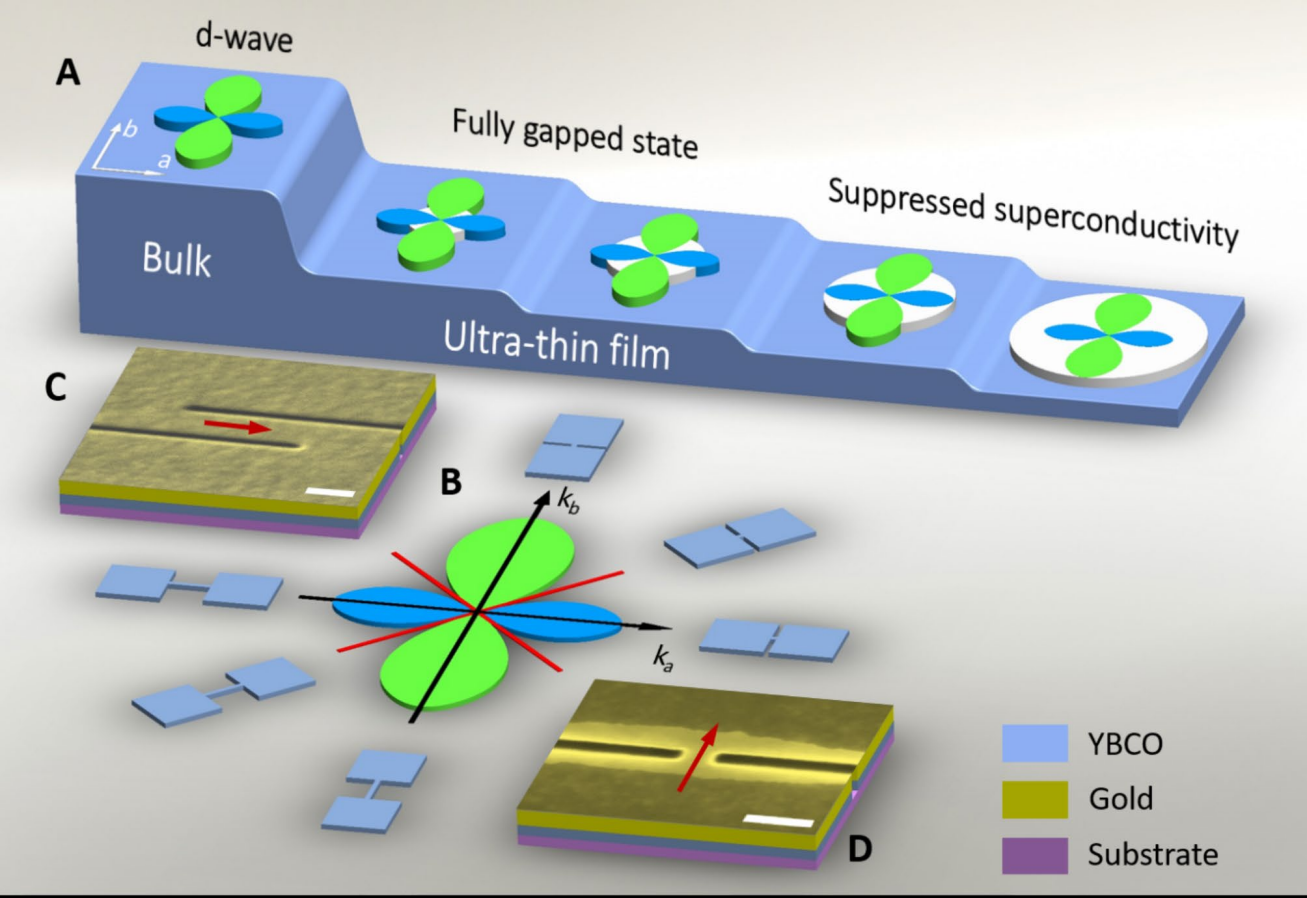
Energy gap in the nanoscale YBCO film. (**a**). Evolution of the energy gap in YBCO with decreasing film thickness. The superconducting energy gap in bulk YBCO is colored in blue and green. The confinement energy gap is colored in white. (**b**). Orientation of the nanowires and nanoconstriction with respect to the YBCO crystallographic axes. Red lines show nodal directions. (**c**), (**d**). SEM micrographs of YBCO nanowire and nanoconstriction. Red arrows show the direction of the current flow. Scale bars correspond to 200 nm.

Conventional low-temperature superconductors are widely used for quantum applications because of an s-wave pairing symmetry where the energy gap exists along the entire Fermi surface providing an exponentially low number of unpaired quasiparticles at low temperatures. High-temperature (high-*T*_*c*_) cuprate superconductors have significantly higher critical temperatures and larger superconducting energy gaps which is beneficial for many applications. However, optimally-doped bulk cuprate superconductors have d_x2−y2_-wave pairing symmetry where the energy gap vanishes in nodal directions^1,2^, as shown in Fig. 1a. The gapless state reduces the significance of high-*T*_*c*_ superconductors for quantum applications because the unpaired quasiparticles in these superconductors exist even at zero temperature.

Nonetheless, there are a number of experimental results including the parity effect in YBa_2_Cu_3_O_7 − x_ (YBCO) nanoparticles^3^, the signatures of s-wave pairing in ultra-thin Bi_2_Sr_2_CaCu_2_O_8 + x_ Josephson junctions^4^ and disordered YBCO films^5^, the single-photon response of the high-*T*_*c*_ nanowires^6,7^, and the high quality factor of YBCO nanowires^8^ that cannot be explained by the d-wave pairing symmetry. The nodeless energy gap has been reported for optimally doped YBCO, underdoped La_2 − x_Sr_x_CuO_4_, and Bi_2_Sr_2–x_La_x_CuO_6+δ_ films^9–11^. Theoretically, it was proposed that the scattering^12^, disorder^13^, or large superconducting phase fluctuations^14,15^ may result in the fully gapped state of the d-wave superconductor. All this evidence gives hope that it is feasible to build fully gapped devices from the high-*T*_*c*_ superconductors, which can be used for quantum applications. However, there is still no recipe for preparing the fully gapped state in the optimally- or overdoped d-wave superconductor.

Following the observation of the fully gaped state in the ultra-thin YBCO nanowires^8^, we fabricate nanowires and nanoconstrictions and use them to investigate the order parameter symmetry in ultra-thin YBCO films and elucidate the nature of the order parameter symmetry transformation. Investigations of the nanoconstrictions and nanowires properties complement each other. Relative values of the energy gap with a high angular resolution can be obtained from the measurements of the nanowire transport characteristics. While the Andreev spectra of nanoconstriction provide information on the absolute magnitude of the energy gap in a wide range of angles.

### Structural properties of ultra-thin YBCO films

**Fig. 2:**
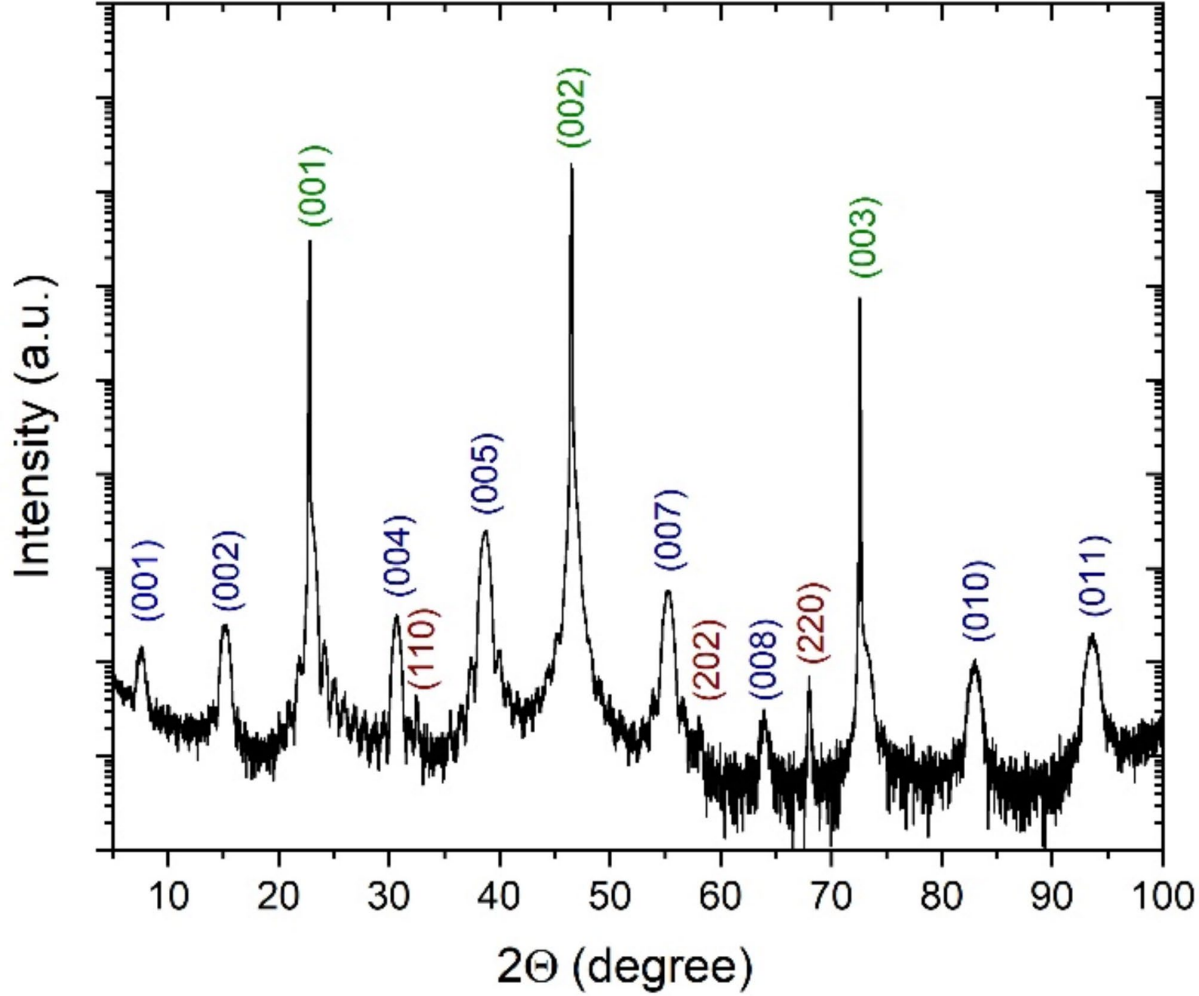
XRD analysis of the ultra-thin YBCO film. High-resolution XRD 2Θ- Θ scan of a 9.9-nm-thick YBCO film capped by a 5-nm-thick amorphous YBCO layer. Peaks corresponding to YBCO film, SrTiO3 substrate, and CuO precipitates are colored in blue, green, and brown, respectively.

Prior fabricating the nanowires and nanoconstrictions, we performed the structural analysis of ultra-thin YBCO films. To investigate the in-plane and out-of-plane crystallographic properties of the ultra-thin YBCO films, we deposited two 9.9-nm-thick YBCO films capped with a 5-nm-thick amorphous YBCO layer on the (100) SrTiO_3_ substrates with the TiO_2_–terminated surface and perform X-ray diffraction (XRD) analysis with a high-resolution Rigaku Smartlab diffractometer. Both samples possess similar results. Figure 2 shows a high-resolution 2θ-θ scan of one of the samples. Only (00X) YBCO reflections are observed in the 2θ-θ scan, indicating single-crystalline growth along the c-axis as the growth direction. Additional reflections in the 2θ-θ scan correspond to SrTiO_3_ substrate and CuO precipitates. An analysis of the scanning-electron microscopy micrographs shows that the precipitates have an average area of 0.45 μm^2^ and occupy 0.36% of the film surface. From the diffraction angles of the (00X) peaks, we calculate the length of the YBCO c-axis parameter as 11.62 Å (Supplementary information, Figure S1) which is shorter than the corresponding table value of 11.68 Å for the optimally-doped YBCO^16^.

**Fig. 3:**
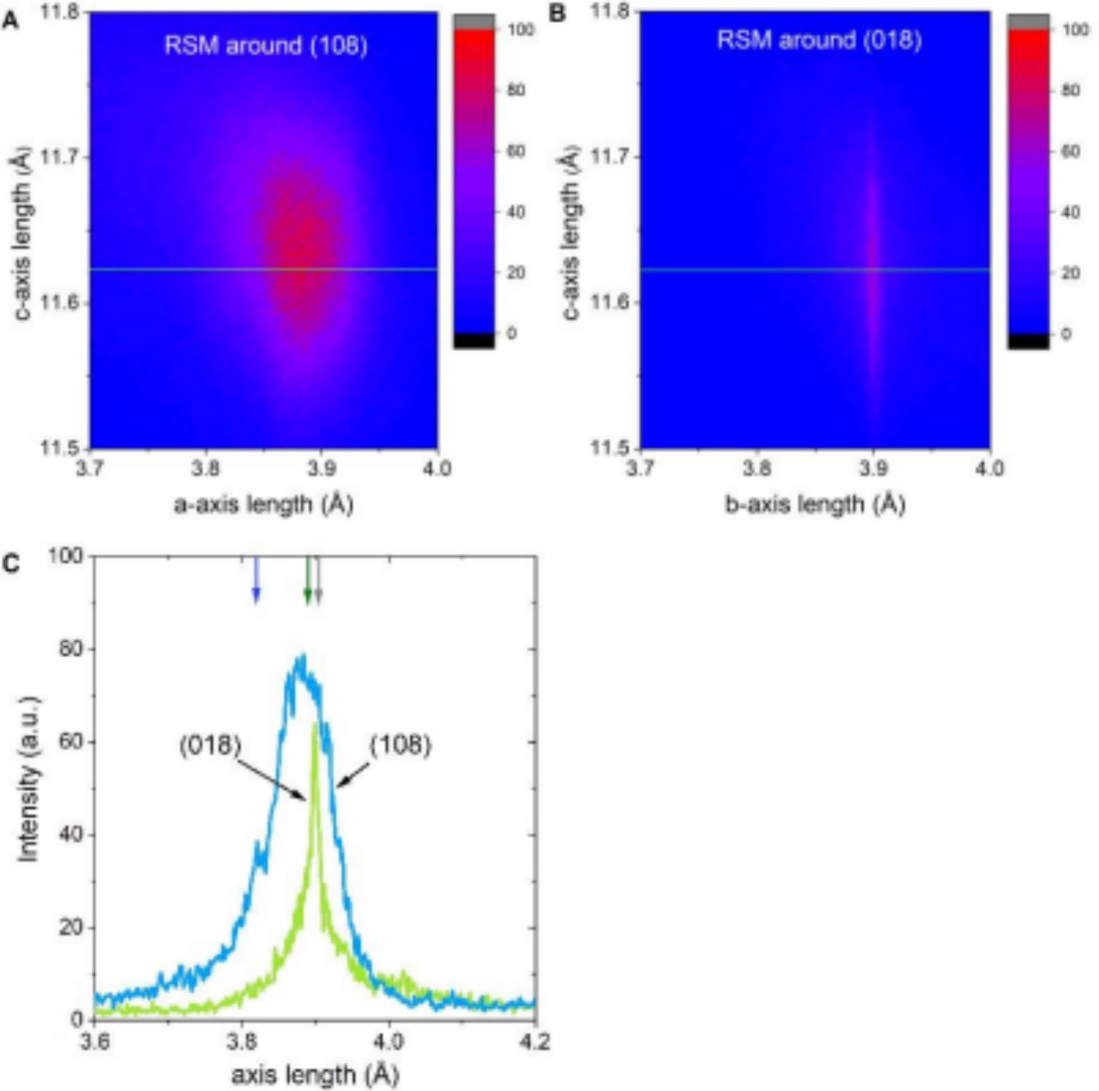
Reciprocal space maps of the ultra-thin YBCO film. High-resolution reciprocal space maps (RSM) of a 9.9-nm-thick YBCO film capped by a 5-nm-thick amorphous YBCO layer around the (**a**) (0,1,8) and (**b**) (1,0,8) reflections. (**c**). Cross sections of RSMs around (108) reflection (light blue line) and (018) reflection (light green line). Blue, green, and grey arrow arrows indicate the table values of the a-axis and b-axis lattice constants of the optimally-doped YBCO and the lattice constant of SrTiO_3_, respectively. Positions of the RSM cross section are shown by green line in figures (**a**) and (**b**).

High-resolution reciprocal space maps (RSMs) around the (0,1,8) and (1,0,8) reflections are shown in Fig. 3a and b. The peak around (0,1,8) reflection is very narrow and differs from the peak around (1,0,8) reflection. We plot the cross sections of the RSMs around the (0,1,8) and (1,0,8) reflections in the Fig. 3c. The values of the lattice parameters determined from the positions on the cross sections peaks are 3.88 and 3.90 Å. These values are longer than the corresponding table values of the lattice parameters of the optimally-doped YBCO, which are **a** = 3.82 and **b** = 3.89 Å, respectively, showing that ultra-thin films are stressed^16^. SrTiO_3_ substrate has a cubic lattice at room temperature with a lattice constant of 3.905 Å. The misfit parameter for the **a**-axis direction of the YBCO is 2.2% while for the **b**-axis direction - only 0.39%. The smaller misfit parameter should correspond to the narrower RSM peak. Therefore, we have adopted the (1,0,8) and (0,1,8) peaks to evaluate the in-plane lattices **a**- and **b**- of the YBCO film, respectively, that are assumed to be ordered along [100] and [010] crystal orientations on the SrTiO_3_ substrate. The RSM peaks corresponding to the **a**- and **b**-axis lattice parameters of YBCO overlap which makes impossible an accurate calculation of the untwining degree.

### Study of order parameter symmetry with nanowires

We fabricate 530-nm-long nanowires with an effective width of 70–100 nm from ultra-thin YBCO films with a thickness *d* in the range of 7–11.6 nm (6–10 unit cells (u.c.)) deposited on a (100) SrTiO_3_ substrate. The YBCO films annealed in oxygen at 800 mbar pressure and temperature of 500 °C have room-temperature Hall coefficient of (7.2 ± 0.1)·10^− 4^ cm^3^/C^17^, which means that the films are overdoped^18^. The c-axis-oriented YBCO films have a roughness of ± 1 u.c^19^. On each substrate, we pattern 13–27 nanowires along the substrate diagonal and edges, which correspond to the nodal and antinodal directions of YBCO, respectively, as schematically shown in Fig. 1b. The nanowires are fabricated in a two-step process. In the first step, 5-µm-wide microbridges oriented along [100] and [010] crystallographic axes of the SrTiO_3_ substrate are patterned using an optical UV contact lithography. The electrical characteristics of the selected microbridges on each substrate are measured before nanopatterning to ensure the film quality.

The films have high critical temperatures and pronounced in-plane anisotropy of the critical current expected for the twin-free films (Supplementary information, Figures S2,S3). In the second step, the nanowires are fabricated across the microbridges with two cuts made with the focused ion-beam milling (FIB). The fabrication process is described in detail in the Methods section. The representative SEM micrograph of the nanowire shaped by FIB two cuts is demonstrated in Fig. 1c. The nanowires are capped with a 15-nm-thick gold layer deposited in situ to get the same boundary conditions as in nanoconstrictions, presented below, where the gold layer prevents overheating at high voltage biases.

**Fig. 4:**
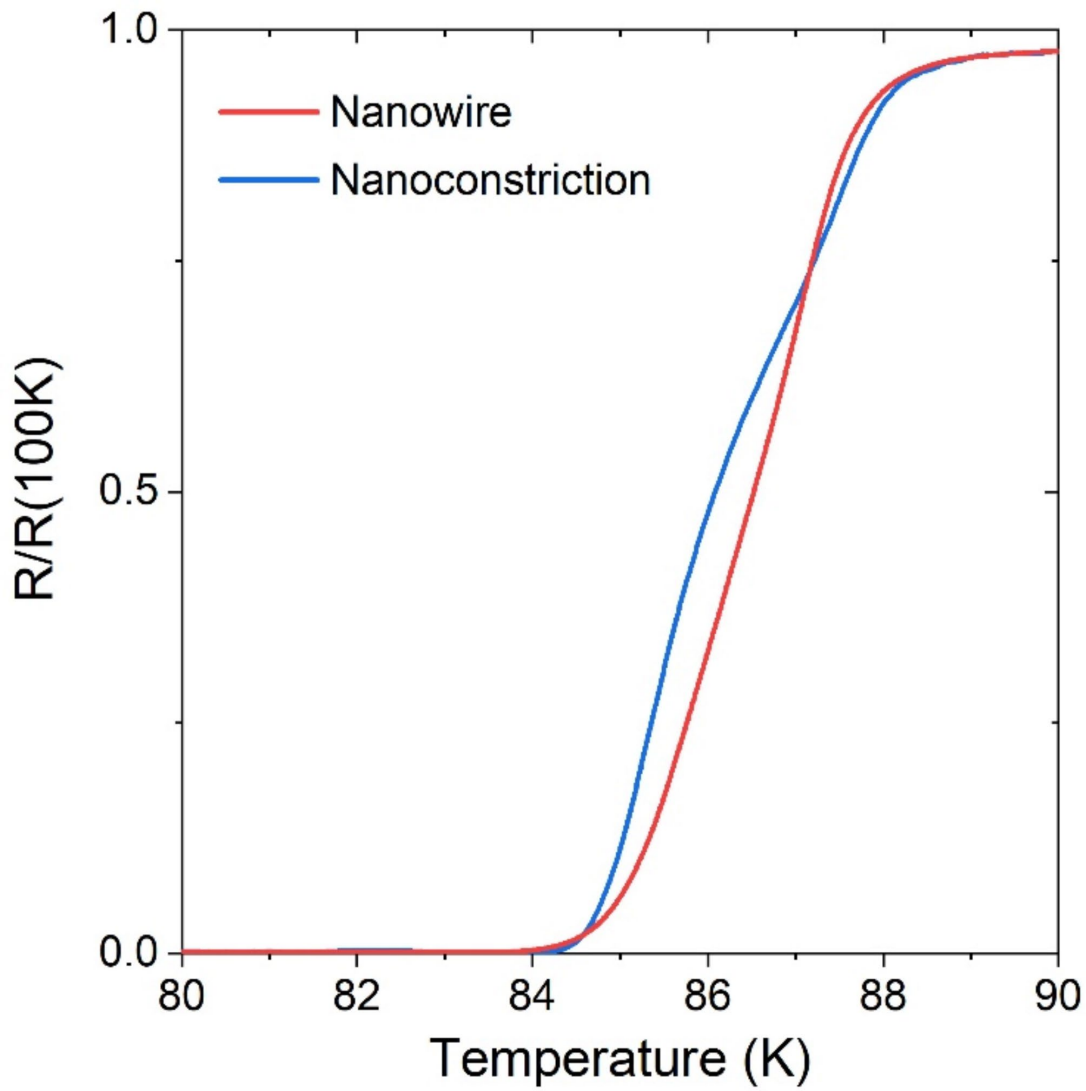
Superconducting transition of the Au/YBCO nanowire and nanoconstriction. Normalized *R*(*T*) curves of the 9-u.c.-thick nanowire (red line) and nanoconstriction (blue line).

The representative temperature dependence of the differential resistance *R*(*T*) of the Au/YBCO nanowire measured in the region of the superconducting transition is shown in Fig. 4. The superconducting transition has a characteristic foot-like behavior, which is weakly expressed in Fig. 4 because the YBCO nanowire is shunted by a gold layer, so that the transition of the electrodes into the superconducting state is barely visible. Transition of the nanowire to the zero-resistance state occurs at the lower temperature compared to the transition of the electrodes because of the thermally-activated phase slippage. The temperature of the superconducting transition of the Au/YBCO nanowires is close to that of the Au/YBCO film (Supplementary Information, Figure S2).

We measure the current-voltage (*IV*) characteristic of the current-biased nanowire at a temperature *T* = 4.2 K and determine the critical current *I*_*c*_ using a 10 µV voltage threshold. For all the nanowire orientations, most *IV* curves demonstrate a voltage switching with a small current hysteresis when the bias current exceeds the critical current (Supplementary information, Figures S4,S5). The voltage switching in ultra-thin YBCO nanowires occurs due to the phase slippage caused by the kinematic vortex motion, as shown in our previous work^20^.

**Fig. 5:**
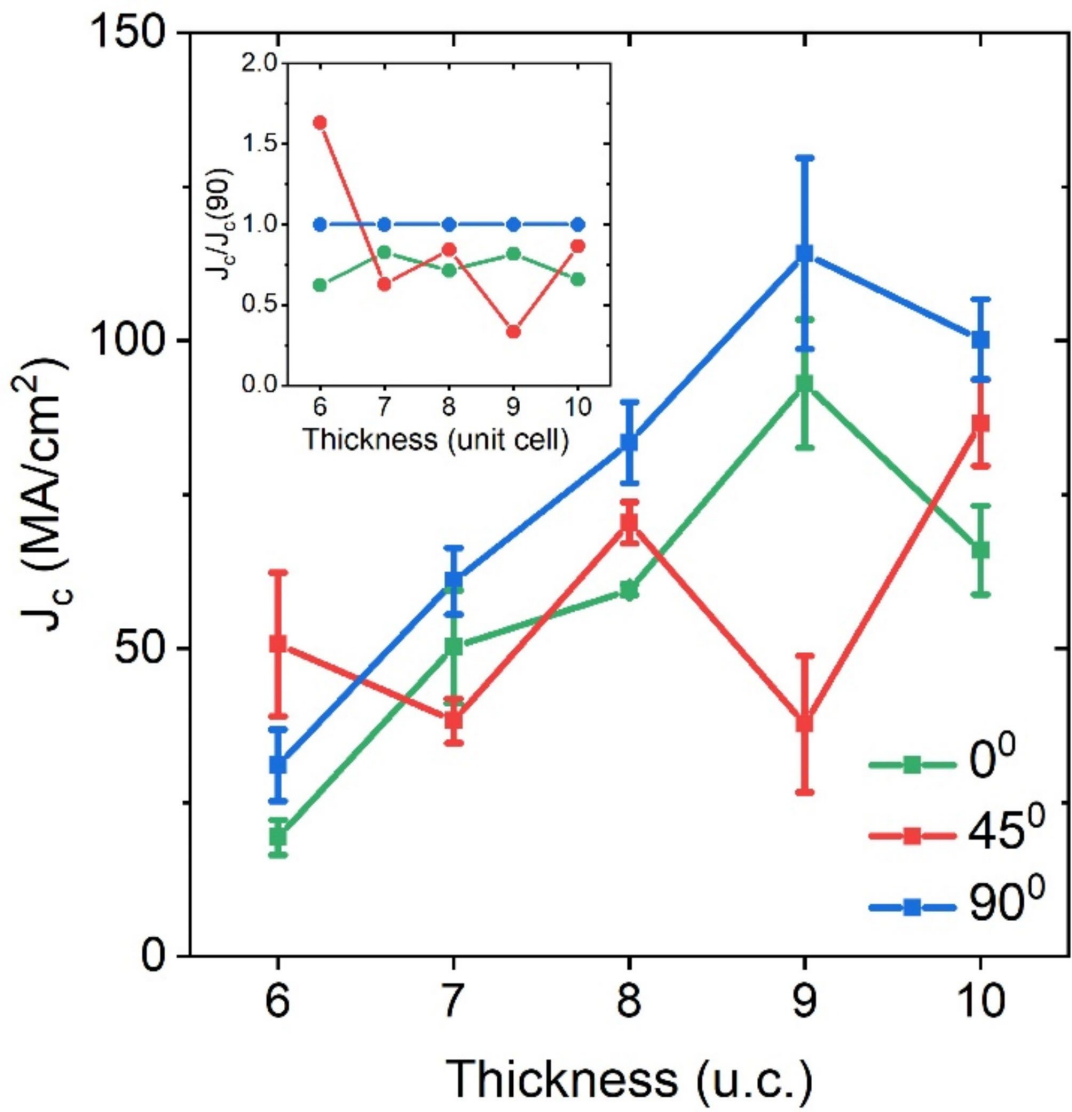
The thickness dependence of the critical current density *J*_*c*_ of the nanowires oriented in different directions with respect to the a-axis at *T* = 4.2 K. Error bars represent one standard deviation. The inset shows the dependence of the normalized critical current density on the nanowire orientation relative to the a-axis.

The kinematic vortices are different from the Abrikosov ones and can be considered as propagating waves carrying the order parameter singularities across the wide superconducting bridge with a width *W* >> *ξ*^21–25^. Here *ξ* is the coherence length. According to Barba-Ortega et al.^21^, the phase slippage in a wide superconducting bridge is expected only in a fully gapped state of a superconductor, as no kinematic vortices exist in a gapless superconductor. The kinematic vortices nucleate in the places where the current density locally reaches the depairing current density and the pairbreaking process leads to a prompt local decrease in the order parameter^24^. The switching to the resistive state in the narrow and ultra-thin YBCO nanowires with high critical current densities occurs after the penetration of a single kinematic vortex^8^. Therefore, in contrast to the superconducting structures, where the critical current is defined by the penetration and pinning of the Abrikosov vortices, the critical current density in the studied YBCO nanowires can be used as an indicator of the order parameter.

We found that an average critical current density of the nanowires oriented along one substrate edge is higher than that of the nanowires oriented along the perpendicular substrate edge as previously observed for the twin-free or partially twinned YBCO films^26^. Therefore, we assign the nanowires with the lower and higher critical current densities to the **a**- and **b**-axis directions, respectively.

The critical current density is calculated as *J*_*c*_=*I*_*c*_/*W*_*eff*_*d*_*eff*_ using the effective width *W*_*eff*_ = *W* – 140 nm instead of geometric width *W*, as outlined in our previous work^20^, and the effective thickness *d*_*eff*_ = *d* -2 u.c. taking into account the non-superconducting YBCO layers at the YBCO film interfaces^19^. We estimate a reduction in critical current density due to current crowding at the ends of the nanowires of less than 0.5% because of the large turnaround radius^27^.

The thickness dependences of the average critical current density < *J*_*c0*_> in the **a**-axis direction (0°), <*J*_*c90*_> in the **b**-axis direction (90°), and < *J*_*c45*_> in the nodal direction (45°) are plotted in Fig. 5. The average critical current density < *J*_*c*_> in the antinodal directions significantly reduces with the decrease of the film thickness preserving the same < *J*_*c0*_>/<*J*_*c90*_> ratio of 0.7 for the 7-10-u.c.-thick nanowires as shown in the inset of Fig. 5. The measured < *J*_*c0*_>/<*J*_*c90*_> ratio is close to the anisotropy of the London penetration depth *λ*_b_/*λ*_a_ and superconducting energy gap in the single-crystal YBCO samples^28,29^.

Remarkably, the thickness dependence of < *J*_*c45*_> (inset of Fig. 5) does not scale with < *J*_*c0*_> or < *J*_*c90*_>, as it would if the symmetry of the bulk order parameter was preserved or the energy gap in the nodal direction had the same origin as the energy gap in the antinodal directions. It also does not show monotonic behavior, as would be expected if the critical current density was affected by surface-related effects (scattering or surface underdoping^30,31^). Instead, <*J*_*c45*_> scatters between 38 and 86 MA/cm^2^, as expected for the size-dependent oscillations of the density of states at the Fermi energy level^32^.

The observation of the voltage switching due to the kinematic vortex motion for all the nanowire orientations together with the theoretical findings of Barba-Ortega et al.^21^ that no kinematic vortices exist in a gapless superconductor provides evidence of the non-zero nodal energy gap in the ultra-thin YBCO films. The nonmonotonic < *J*_*c45*_> thickness dependence indicates that the origin of the nodal gap differs from that of the intrinsic energy gap in YBCO.

### Study of origin of the energy gap with nanoconstrictions

After getting evidence of the non-zero energy gap in the nodal directions, we now turn to the question of the absolute value of this gap. To answer this question, we fabricate nanoconstrictions from 5-9-u.c.-thick YBCO films and measure their electrical transport characteristics in the 4.2–90 K temperature range. On each substrate, we pattern 7 or 13 nanoconstrictions in the nodal and antinodal directions, as schematically shown in Fig. 1b. As the nanowires, the nanoconstrictions are fabricated in a two-step process which is described in detail in the Methods section.

At currents above the critical current, a superconducting constriction can be modeled as a superconductor-normal metal-superconductor (SNS) junction where the voltage is developed across the dissipative neck region^33,34^. Within the framework of the simplified theoretical approach to SNS junctions, each quasiparticle undergoes multiple Andreev reflections (MAR) before it scatters or leaves the pair potential well. If a quasiparticle undergoes *n* Andreev reflections (AR), then *ne* charges are transferred through the NS boundary in addition to the initial one, and the SNS junction current is enhanced due to AR. Here *n* is an integer and *e* is an electron charge. As a consequences of MAR, the *IV* curves and especially the conductance curves show many nonlinear structures^35–37^.

At higher voltages *V*$\:\gtrsim\:$ Δ/2*e*, the number of AR becomes energy limited, decreasing with increasing energy that the quasiparticle received per AR and only a few isolated dips occur in the conductance curve^37^. In this high-voltage region, each current enhancement due to AR leads to the corresponding step-wise increase of the SNS junction conductance. According to these theoretical predictions, the measurements of the AR spectrum provide information on the magnitude of the superconducting energy gaps in the nanoconstriction electrodes^34–38^.

**Fig. 6:**
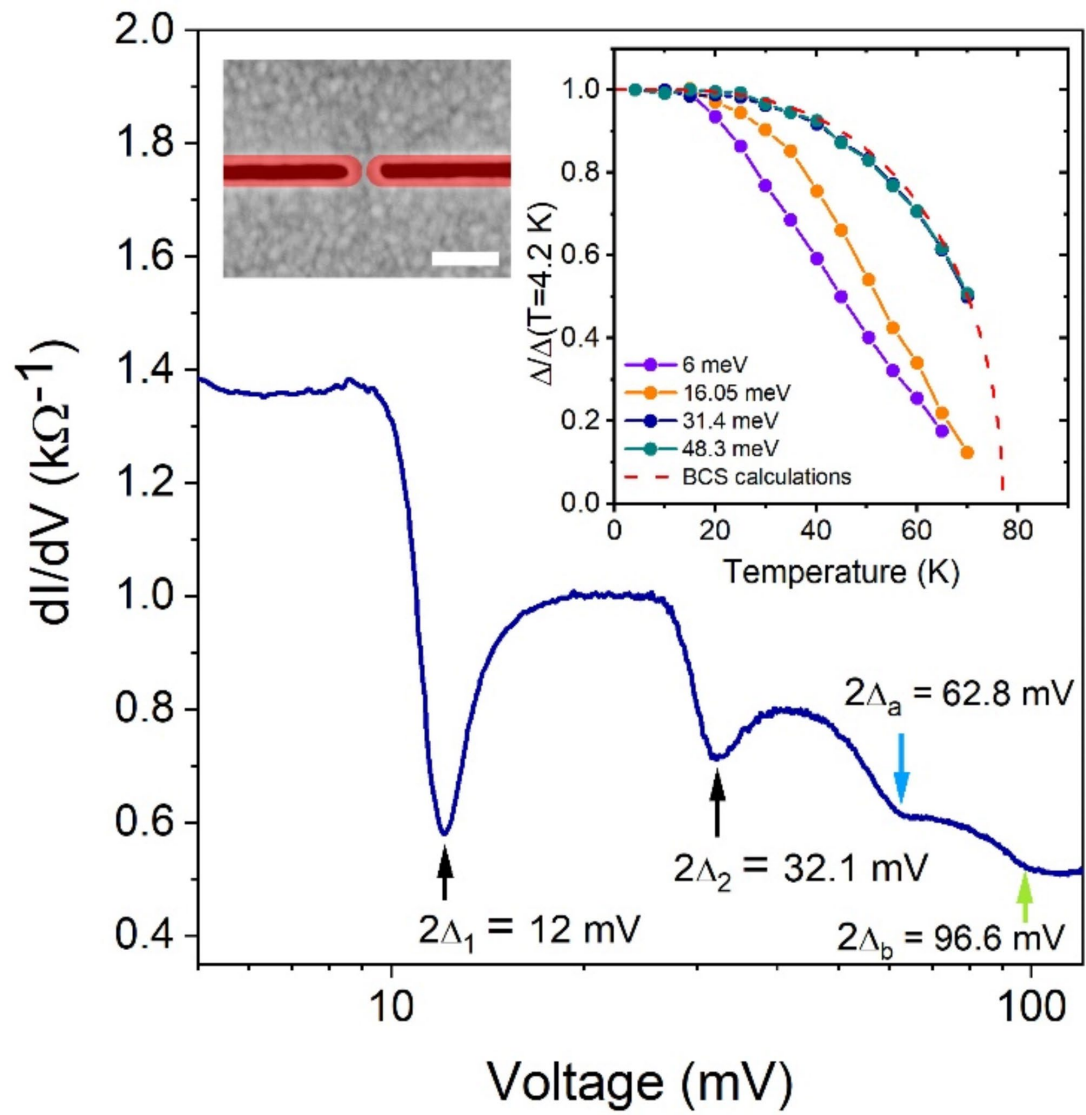
Differential conductance of the 6-u.c.-thick ultra-narrow YBCO nanoconstriction at 4.2 K. Blue, green, and black arrows indicate the position of the conductance steps. Insets: left. SEM image of the ultra-narrow nanoconstriction just after the FIB milling. The non-superconducting YBCO is colored in red. The scale bar corresponds to 200 nm. Right. the temperature dependences of the normalized energy gaps. The dashed line shows the BCS theory prediction.

The YBCO has large superconducting energy gaps^28^ that make the conductance measurements up to voltages *V* ≥ 2Δ/*e* ≈ 90 mV challenging because of the constriction overheating. First, we use FIB milling (see Methods) to fabricate ultra-narrow constrictions with very high resistances from bare YBCO films that do not show overheating even at voltages above 100 mV. The YBCO films are capped in situ with the 6-nm-thick insulating amorphous YBCO layer^19^ to prevent the film degradation during the fabrication. The number of such ultra-narrow constrictions with a measurable critical current was limited by several samples because of a low fabrication yield. The differential conductance *dI/dV* and the SEM micrograph of the high-resistance 6-u.c.-thick YBCO nanoconstriction with the *I*_*c*_ = 11 µA at *T* = 4.2 K are presented in Fig. 6 and the left inset of Fig. 6, respectively. The nanoconstriction is oriented in the antinodal direction. The estimated width of this constriction is 3–6 nm which is comparable to the a-b plane coherence length of YBCO *ξ*_*ab*_ = 1.3–1.6 nm^18,39^. The *dI/dV* curve has a well-defined ladder-like structure due to AR in the normal-state region of the nanoconstriction^34,37,38^. We exclude the appearance of the conductance steps due to the multiple phase-slip centers or flux-flow instabilities. The nanoconstrictions are significantly shorter than the electric field penetration depth in YBCO^20^ to accommodate multiple phase-slip centers. The voltage at which the flow instability occurs decreases with decreasing temperature^20,40^, while the temperature dependence of the conductivity steps of the YBCO nanoconstriction is opposite.

The sharpness and shape of the AR features depend on many parameters including scattering, constriction length, quasiparticle mean-free path, the broadening of the coherence peaks of the density of states with increasing anisotropy of the superconducting energy gap, and others. These properties are well explained within the framework of Kümmel, Gusenheimer, and Nikolsky’s nonequilibrium microscopic theory^35^ originally developed for transport in isotropic s-wave SNS junctions and which we extend to anisotropic superconducting pairing^41^. We solve the time-dependent Bogoliubov-de Gennes equations which are combined with the relaxation time model for charge transport, through which the mean free path for inelastic scattering $\:l$ enters into calculations. The anisotropic energy gap is modeled as Δ(*Θ*) = Δ(1 + 0.5*A*[cos(4π*Θ*)-1]), where $\:A$ is the coefficient reflecting the gap anisotropy, $\:\varDelta\:\:$being the maximum value of Δ(*Θ*) for all considered anisotropic cases, while *Θ* is the angle the quasiparticle momentum with respect to the NS interface normal. Details of the numerical calculations can be found in Refs. 35 and 41. Due to the angular dependence of the order parameter, MAR would not be allowed for quasiparticle momenta directions corresponding to the positions of the gap nodes. Therefore, nonlinearities on CVC corresponding to MAR will result only from a subset of momenta directions broadening the sharp structures occurring in the isotropic s-wave case. Our numerical simulations show that for the given barrier properties, the sharpness, and the shape of the conductance step are an indicator of the energy gap anisotropy in the electrodes (Supplementary information, Figure S6). The sharper the step the lower the anisotropy.

The positions of the smeared steps at voltages *V* ≈ 62.8 mV and 96.6 mV shown by blue and green arrows are very close to the expected values of 2Δ/e corresponding to the highly-anisotropic Δ_a_ and Δ_b_ energy gaps in the **a**- and **b**-axis directions in the optimally-doped bulk YBCO, respectively^28^. Their temperature dependences overlap and are close to the prediction of the Bardeen–Cooper–Schrieffer (BCS) theory, as shown in the right inset of Fig. 6 (the *dI/dV* dependencies measured in the 4.2–80 K temperature range are available in the Supplementary information, Figure S8). The simultaneous presence of the conductance steps originating from the energy gaps in both antinodal directions can be explained by the rounded NS surface arising in the ultra-narrow constriction due to the self-heating^42^.

The conductance steps at 12 mV and 32.1 mV have larger amplitude and different temperature dependence compared to those at *V* = 2Δ_a_/e, 2Δ_b_/e. The temperature dependencies of the current position of these steps are different from that of the critical current, as shown in the Supplementary information (Figure S9), giving confidence that these features are not critical current features. These steps are sharper than the conductance steps at *V* ≈ 62.8 mV and 96.6 mV and have a well-defined dip. Therefore, the corresponding energy gaps with Δ_1_(4.2 K) = 6 meV and Δ_2_(4.2 K) = 16.05 meV have smaller anisotropy compared to Δ_a_ and Δ_b_ energy gaps, as follows from the comparison with the results of the numerical simulations presented in the Supplementary information (Figure S6). The Δ_1_(*T*) dependence has a characteristic slope change at about 50 K, which can be explained by assuming that this region of YBCO has lower *T*_*c*_ and is proximetized by the YBCO with Δ_a_ = 31.4 meV and Δ_b_ = 48.3 meV. Therefore, we assume that the energy gap Δ_1_(4.2 K) = 6 meV belongs to the underdoped YBCO neighboring the nanoconstriction neck. The sharp increase in the critical current at *T* ≤ 10 K (Supplementary Information, Figure S9) can be due to the transition of the underdoped region into the superconducting state at a lower temperature compared to the electrodes. The critical temperature of the Δ_2_(*T*) dependence obtained from the linear extrapolation of the Δ_2_(*T*) dependence is close to the critical temperature of the YBCO with Δ_a_ = 31.4 meV and Δ_b_ = 48.3 meV determined from the BSC theory fitting (dashed line in the inset of Fig. 6). Therefore, we assume that the energy gaps with Δ_2_(4.2 K) = 16.05 meV, Δ_a_ = 31.4 meV, and Δ_b_ = 48.3 meV appear in the same region of YBCO. In addition, there is no sharp increase in the critical current that could be associated with the transition of the separated region of YBCO with Δ_2_(4.2 K) = 16.05 meV into the superconducting state. AR spectra of the ultra-narrow constrictions provide clear evidence of new energy gaps in ultra-thin YBCO films that are not inherent to the bulk YBCO. However, the current in the ultra-narrow nanoconstrictions may flow through the oxygen-depleted YBCO regions which complicates the analysis.

To overcome this problem, we measure additionally 100-nm-wide and 40-50-nm-long YBCO nanoconstrictions covered in situ by a 15-20-nm-thick gold layer. The fabrication process is described in Methods. The 100-nm-wide constrictions are wide enough to avoid electrical transport across the degraded YBCO in the constriction area. The gold layer removes the heat from the constriction area and prevents the normal-state region in the constriction from expanding. These Au/YBCO nanoconstrictions are fabricated with electron-beam lithography and ion-beam etching, as described in the Methods. The Au/YBCO nanoconstrictions are oriented along the substrate diagonal and edges, which correspond to the nodal and antinodal directions of YBCO, respectively, as schematically shown in Fig. 1b. A representative SEM micrograph of the nanoconstriction is shown in Fig. 1d.

The representative *R*(*T*) curve of the 9-u.c.-thick Au/YBCO nanowire in the superconducting transition region is shown in Fig. 2. As for Au/YBCO nanowires, the superconducting transition has a characteristic foot-like behavior. In nanoconstrictions, this behavior is more pronounced because their resistance is lower. The temperature of the superconducting transition of the Au/YBCO nanoconstrictions is close to that of the Au/YBCO film (Supplementary Information, Figure S2).

**Fig. 7:**
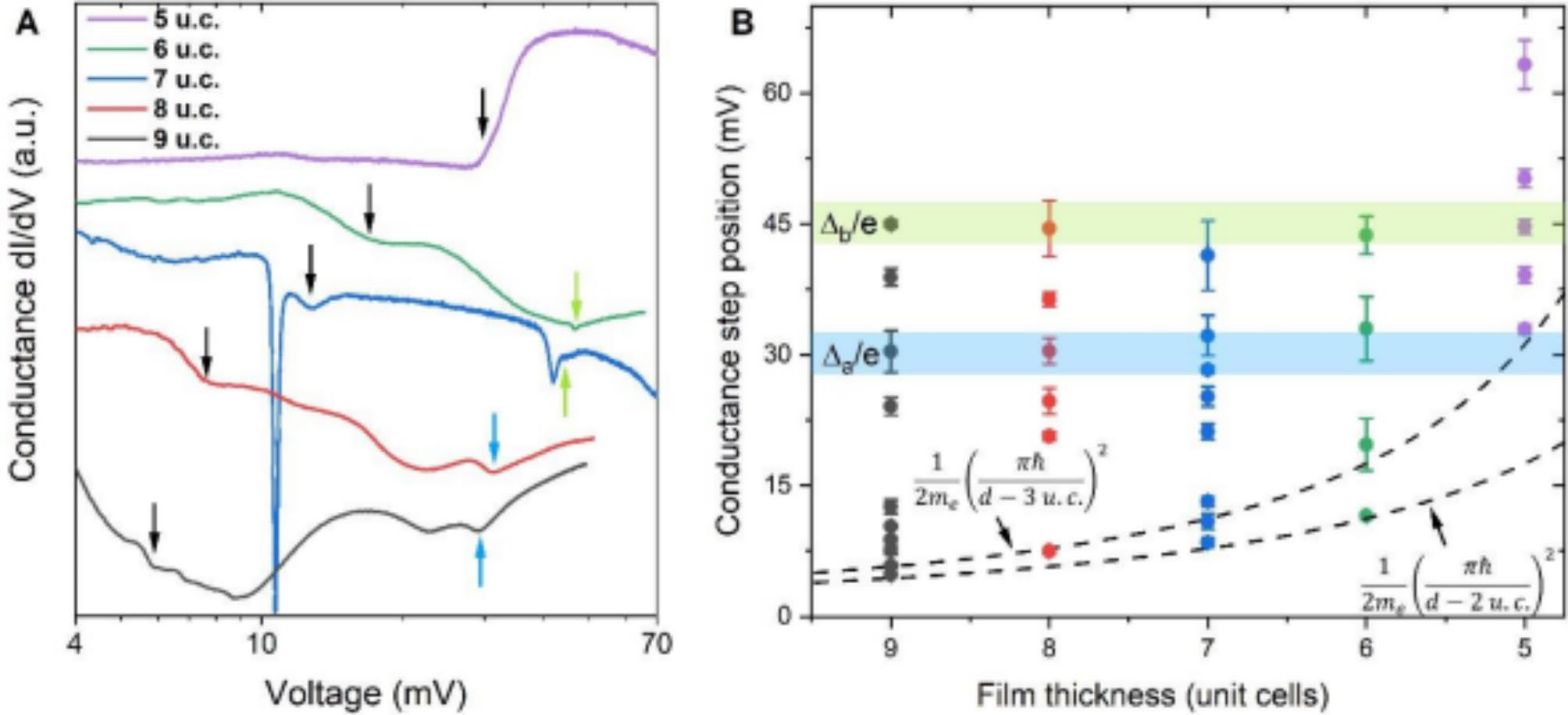
Energy gaps in ultra-thin YBCO films of various thicknesses. (**a**). Representative differential conductance of 100-nm-wide Au/YBCO nanoconstrictions of various thicknesses. Curves are shifted along the Y-axis for convenience of their presentation. Blue, green, and black arrows indicate positions of the conductance steps induced by Δa, Δb, and energy gap not inherent to bulk YBCO, respectively. (**b**). Thickness dependence of the positions of the conductance steps for the Au/YBCO nanoconstrictions. Error bars represent one standard deviation.

Representative differential conductance curves of 100-nm-wide and 5–9-u.c.-thick Au/YBCO nanoconstrictions are shown in Fig. 7a. Except for the thinnest nanoconstrictions, the differential conductance demonstrates steps similar to those observed for the ultra-narrow constriction without gold capping layer. We clearly identify the conductance steps at high voltages as corresponding to the intrinsic YBCO energy gaps because they are observed for most nanoconstrictions and consistent with the expected antinodal energy gaps of YBCO^28^. These steps, which appear at voltages *V* = Δ_a_/e ≈ 30 mV and *V* = Δ_b_/e ≈ 45 mV, are indicated in Fig. 7a by blue and green arrows, respectively. In addition, we observe conductance steps at lower voltages indicated in Fig. 7a by black arrows. Some of the conductance steps are accompanied by very sharp dips as in the case of the 7-u.c.-thick nanoconstriction. These sharp dips can occur in the current bias regime due to the S-type nonlinearity near the *V* = 2Δ/*ne*, as shown in the Supplementary information (Figure S7). The conductance of the 5-u.c.-thick nanoconstrictions is almost constant below the voltage of 30 mV and then increases sharply. This behavior is qualitatively different from the Andreev-reflection-like type of conductance observed for thicker nanoconstrictions. In addition to the changes in the conductance behavior, the critical current density of the 5-u.c.-thick nanoconstrictions decreased by one order of magnitude in one antinodal direction and by two orders of magnitude in the other antinodal and nodal directions compared to that of the thicker nanoconstrictions.

We plot the positions of the steps found at the differential conductance of the seventy 100-nm-wide Au/YBCO nanoconstrictions of various thicknesses in Fig. 7b. The region corresponding to the Δ_a_/e and Δ_b_/e of the untwinned YBCO samples^28^are colored in blue and green, respectively. The conductance curves of the almost all Au/YBCO nanoconstrictions have steps at the voltages which are close to the Δ_a_ and Δ_b_ of the untwinned YBCO samples. There are also steps at voltages about 20 mV. However, an accurate assignment of these conductance steps either to 2Δ_a_/ne series or 2Δ_b_/ne series is difficult because Δ_b_ ≈ 1.5Δ_a_ in YBCO and a part of MAR harmonics originating from Δ_a_ intersects with a series of MAR harmonics originating from Δ_b_. The conductance steps at *V* = 20 mV can be referred to either 2Δ_a_/3e or Δ_b_/2e. However, the low-voltage conductance steps shown in Fig. 7a by black arrows belong neither to 2Δ_a_/*ne* series nor to 2Δ_b_/*ne* series. These conductance steps are caused by the energy gaps which are not inherent to the bulk YBCO. The energies of these low-energy gaps are smaller for the thicker films and higher for the thinner ones. The positions of the conductance steps are limited from below by (1/*d*)^2^-type dependence which is shown in Fig. 7b by dashed lines.

### Quantum size effects in nanoscale YBCO films

The (1/*d*)^2^-type dependence of the energy gap on the device size is typical for the quantum size effects (QSE) which occur when at least one dimension of the device is comparable with the Fermi wavelength *λ*_*F*_ = *h*/*mv*_*F*_, where *h* is the Planck constant, *m* is the charge carrier mass, and *v*_*F*_ is the Fermi velocity. QSE are well known for semiconducting nanodevices because of the large Fermi wavelength^43^. The observation of QSE in metals is more challenging because of their very short Fermi wavelength *λ*_*F*_ ≈ 5 Å. QSE can induce a pseudo gap and change the conductivity type of the atomically-flat nanoscale semimetal^44–46^ and metal films^47–49^ from metallic to semiconducting. A change in the density of states in the nanoscaled BCS superconductors due to the QSE results in the change in a superconducting energy gap value and, consequently, in the change in the critical temperature^32,50–56^.

The Fermi velocity in high-*T*_*c*_ superconductors is an order of magnitude lower than in metallic superconductors resulting in a larger Fermi wavelength. We estimate the Fermi wavelength in YBCO as *λ*_*F*_(YBCO) ≈ 2 nm using *m* ≈ 2*m*_*e*_^57,58^ and *v*_*F*_ = 2·10^5^ m/Sec^59^, which is comparable with the total thickness of studied films *d* = 5.8–10.5 nm. Therefore, the superconducting properties of our ultra-thin YBCO films which have very low surface roughness have to be affected by QSE.

QSE modify the magnitude of the superconducting energy gap in s-wave superconductors but do not change the order parameter symmetry. In contrast to s-wave superconductors, QSE have a more significant effect on the properties of d-wave superconductors. The bulk d-wave superconductors have nodes where the superconducting energy gap vanishes, as shown in Fig. 1a. An opening of the energy gap in the nodal direction, as it is illustrated in a simplified way at the second and third steps from the left side in Fig. 1a, brings YBCO into the fully gapped state with an exponentially low number of quasiparticles at low temperatures.

The thickness dependence of the new gap in the ultra-thin YBCO films is in good quantitative agreement with the well-known expression for the confinement energy in the quantum well *E*_*g*_ = [*ħπ*/*d*]^2^/2*m* (1) which is shown in Fig. 7b by dashed lines. We plot two *E*_*g*_-dependencies which account for the film roughness of 1 u.c. and nonsuperconducting layer at the YBCO-substrate interface. The upper and the lower dashed lines correspond to *E*_*g*_ = [*ħπ*/(*d-*3 u.c.)]^2^/2*m*_*e*_ and *E*_*g*_ = [*ħπ*/(*d-*2 u.c.)]^2^/2*m*_*e*_, respectively.

The confinement gap does not strongly affect the critical current density of the 6-10-u.c.-thick films, where its magnitude is relatively small. But the changes in the superconducting properties become more drastic when the magnitude of the confinement gap approaches the value of the superconducting energy gap, as shown at the rightmost steps in Fig. 1a. The superconducting order parameter is strongly dependent on the number of the single-electron states inside the Debye “window” around the Fermi level^60^. The increasing confinement energy gap first reduces the number of states that can be used for the quasiparticle pairing resulting in the decrease of the critical current density, as it is confirmed by the experimental results for the nanowires in Fig. 5. When the magnitude of the confinement gap approaches the value of the Debye energy, which is ħω_D_ ≈ 40 meV ≈ Δ_b_ in YBCO^61^, the critical current density is strongly suppressed and the conductance behavior is dominated by the confinement energy gap as it is observed for the YBCO nanoconstrictions with the total and effective thickness of 5 and 3 u.c., respectively.

Qualitatively, the physical picture of the energy gap of ultra-thin YBCO films at low temperatures is similar to the model of a narrow-band-gap semiconductor with a local attraction between the carriers introduced by Nozières and Pistolesi^62^. The single particle excitations in such a system are always gapped with the gap equal to max[E_g_, Δ]. Superconductivity in such a system exists until the gain in interaction energy due to superconductivity can overcome the cost of kinetic energy in producing free carriers across the non-superconducting gap. When the magnitude of the non-superconducting energy gap becomes large enough, a superconductor-semiconductor transition occurs, which is in agreement with our experimental findings for 5-u.c.-thick nanoconstrictions.

The fully gapped state due to QSE explains many unusual experimental results with nanoscale cuprate superconductors which contradict the d-wave symmetry. Finally, we compare our findings with the preceding experimental results. In our previous work, we estimated the superconducting energy gap for 4-u.c.-thick nanowire as 17 meV^8^ which is in excellent agreement with the Eq. (1). Gustafsson et al.^3^ reported on the fully gapped state of the 200 × 200 × 100 nm^3^ YBCO nanoparticle with Δ = 18 µeV at zero magnetic field which is again in good quantitative agreement with *E*_*g*_ = 10–40 µeV calculated with Eq. (1). The increase of the superconducting energy gap of YBCO nanoparticle in strong magnetic field observed by Gustafsson et al.^3^ can be explained by the increase of the confinement energy gap in strong magnetic fields^63^.

## Conclusions

We investigate the superconducting properties of ultra-thin YBCO films and find that quantum size effects open the energy gap in the nodal direction and turn the d-wave superconductor into the fully gapped state with the magnitude of the nodal energy gap given by the confinement energy *E*_*g*_ = (*ħπ*/*d*)^2^/2*m*_*e*_. The nanoscale YBCO film can be considered as a quantum-engineered superconductor where the superconducting gap is controlled by quantum effects. The fully gapped state paves the way for nanoscale d-wave superconductors towards many quantum applications including quantum computing and single-photon detection.

## Methods

### Nanostructure fabrication

YBCO nanowires and nanoconstrictions were fabricated from a 5.9–11.6 nm (5–10 unit cell) thick epitaxial YBCO films deposited on a TiO_2_-terminated (100) single-crystal SrTiO_3_ substrate by dc sputtering at high (3.4 mbar) oxygen pressure and substrate heater temperature of 950 °C. One unit cell corresponds to the YBCO lattice parameter in **c**-direction which is 11.62 Å. Substrate edges are aligned along (010) and (001) crystallographic planes. The film thickness was controlled by the deposition time. The deposition rate of 1.0 nm/min was measured by XRR and atomic-force microscope measurements. After the deposition, the films were annealed in oxygen at a pressure of 800 mbar and substrate heater temperature of 500 °C. The YBCO film deposition is described in more detail elsewhere^19^. After the annealing, YBCO films were in situ capped either with a 6-nm-thick amorphous YBCO layer deposited at room temperature in the case of the ultra-narrow nanoconstrictions or by the 15–20 nm-thick gold layer deposited at *T* = 100 °C by magnetron dc sputtering in argon at a pressure of 5·10^− 3^ mbar. We do not see any effect of the gold layer on the nanowire or nanoconstriction superconducting properties. The 100-nm-thick Au contact pads were deposited *ex situ* using room temperature dc magnetron sputtering with a shadow mask. Following contact pad deposition, the nanostructures were fabricated in a two-step process. In the first step, 5-µm-wide microbridges oriented along [100] and [010] crystallographic axes of the SrTiO_3_ substrate were patterned using an optical UV contact lithography with a PMMA resist and wet chemical etching in a Br-Ethanol and I_2_-NaI-Ethanol solutions. Optical micrographs and electrical parameters of the microbridges are available in the Supplementary Information (Figure S10). The electrical characteristics of the selected microbridges on each substrate were measured before nanopatterning to ensure the film quality. In the second step, thirteen 530-nm-long Au/YBCO nanowires or ultra-narrow high-resistance YBCO nanoconstrictions capped with the amorphous YBCO layer were fabricated across the microbridges with two cuts made with FIB milling using an Au/PMMA protective layer. In order to increase the number of nanowires that can be placed at the same substrate and measured with the existing contact system, we also pattern additional fourteen nanowires across the current leads. The Au/PMMa protective layer was removed in acetone before electrical measurements. The width of each nanowire was measured using SEM. More details on the FIB patterning process can be found in Ref. 20. The 100-nm wide and the 40-50-nm-long Au/YBCO nanoconstrictions were made using the inverse process. In the first step, the nanostructures were fabricated using the ion-beam etching in argon through the 80-nm-thick CSAR62 resist mask patterned by the electron-beam lithography. In the second step, the microbridges were defined by an optical UV contact lithography and wet chemical etching using the alignment markers fabricated during the first step. In total 158 nanowires and more than 75 nanoconstrictions have been fabricated.

### Experimental setup

The experimental setup was based on a liquid helium storage Dewar insert filled with He exchange gas. The temperature above 4.2 K was maintained with the resistive heater controlled by a Lake Shore temperature controller 335. We used battery-operated low-noise analog electronics to sweep the bias current and amplify the voltage across the nanostructure. The differential resistance of the nanostructure was measured with lock-in amplifier 7265 (Signal Recovery) at 10 kHz modulation frequency.

## Electronic supplementary material

Below is the link to the electronic supplementary material.

Supplementary Material 1

## Data Availability

The source data for Figs. 4, 5, 6 and 7 is available on Jülich DATA Repository https://data.fz-juelich.de/dataset.xhtml? persistentId=doi:10.26165/JUELICH-DATA/25OXIU.
