# Supplementary material for: Quantum size effects in ultra-thin YBa2Cu3O7 − x films
Source: Sci Rep. 2024 Sep 27;14:22174. doi: 10.1038/s41598-024-73207-z (PMC11436648; doi:10.1038/s41598-024-73207-z)
Supplement: Supplementary file 1 — Supplementary Material 1 [file 41598_2024_73207_MOESM1_ESM.docx]

**Supplementary information**

**Quantum size effects in ultra-thin YBa_2_Cu_3_O_7-x_ films**

M. Lyatti^1,2*^, I. Gundareva^1,2^, T. Röper^1,2^, Z. Popović ^3^, A.R. Jalil^2,4^, D. Grützmacher^1^, T. Schäpers^1,2^

^1^ Peter Grünberg Institut (PGI-9), Forschungszentrum Jülich, 52425 Jülich, Germany

^2^ JARA-Fundamentals of Future Information Technology, Jülich-Aachen Research Alliance, Forschungszentrum Jülich and RWTH Aachen University, Germany

^3^ University of Belgrade, Faculty of Physics, Studentski trg. 12, 11001 Belgrade, Serbia

^1^ Peter Grünberg Institut (PGI-10), Forschungszentrum Jülich, 52425 Jülich, Germany

| **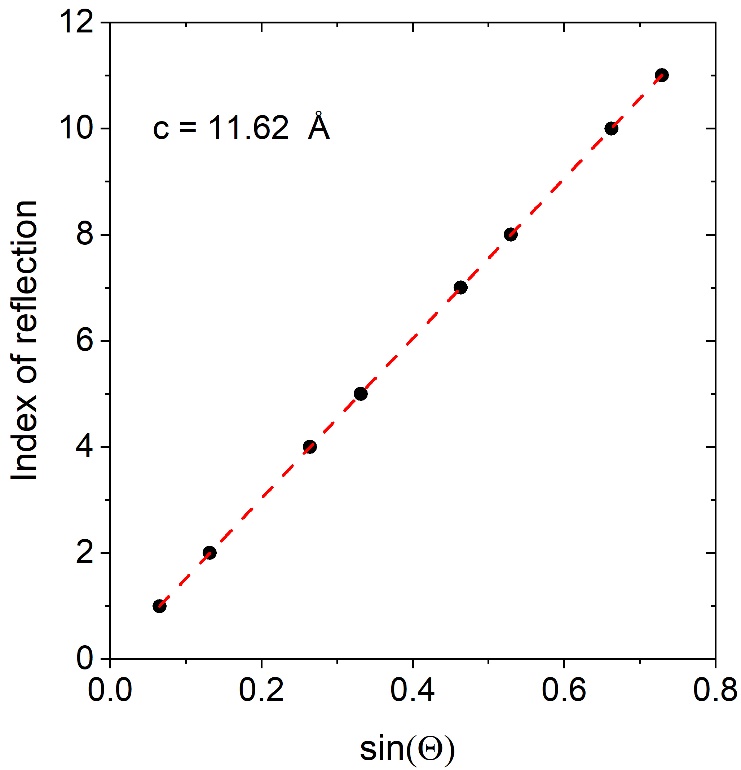** |
| --- |
| **Figure S1\|** YBCO film c-axis parameter obtained by the linear fitting of the (00X) reflections positions. |

| 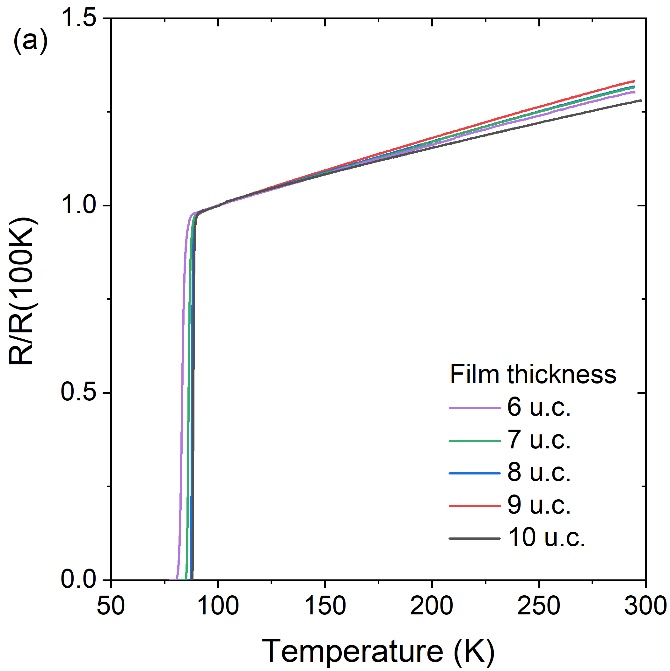 | 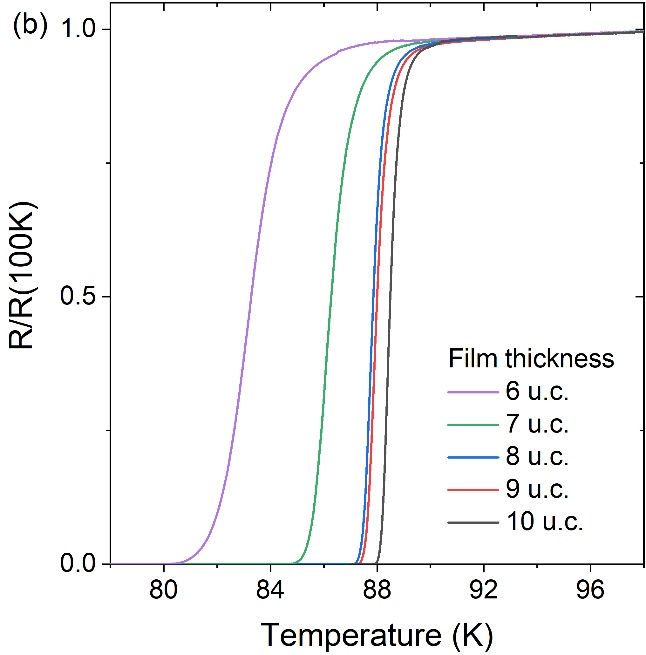 |
| --- | --- |
| **Figure S2\|** a. Temperature dependence of the normalized resistance of the 5-μm-wide Au/YBCO microbridges of various thicknesses. b. Zoomed temperature region of the superconducting transition of the Au/YBCO microbridges. | |

| 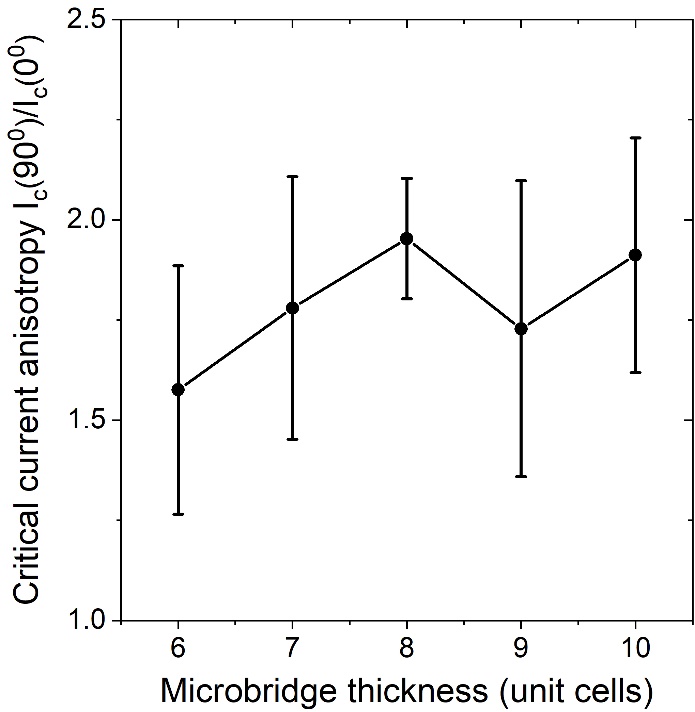 |
| --- |
| **Figure S3\|** Anisotropy of the average critical current density <J_c90_/J_c0_> of the 5-μm-wide Au/YBCO microbridges of various thicknesses measured at temperature of 77.4 K. |

| 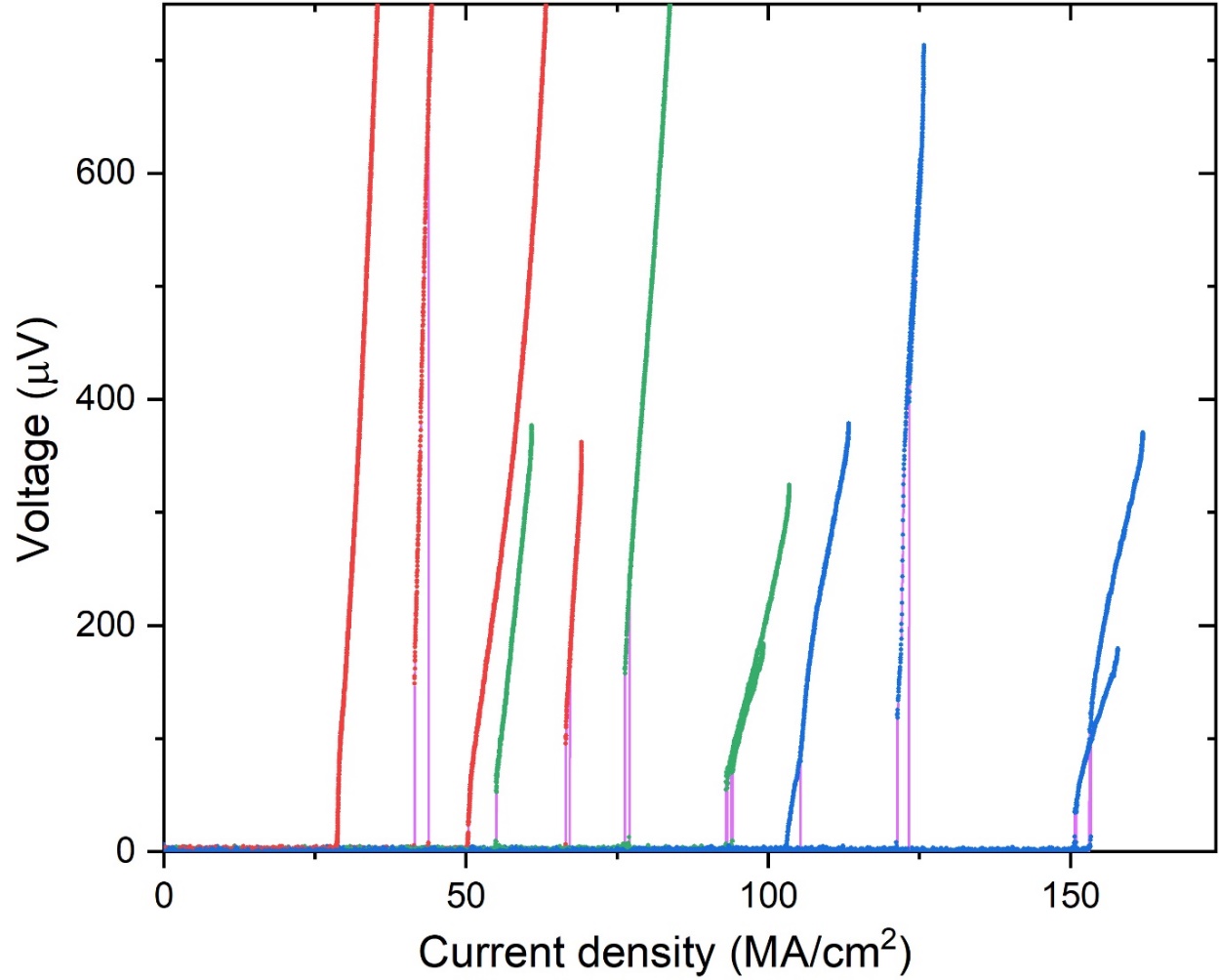 |
| --- |
| **Figure S4\|** *IV* curves of twelve 9-u.c.-thick and 530-nm-long Au/YBCO nanowires at *T* = 4.2 K. *IV* curves of the nanowires oriented along a-axis, b-axis, and nodal directions are colored in green, blue, and red, respectively. |

| 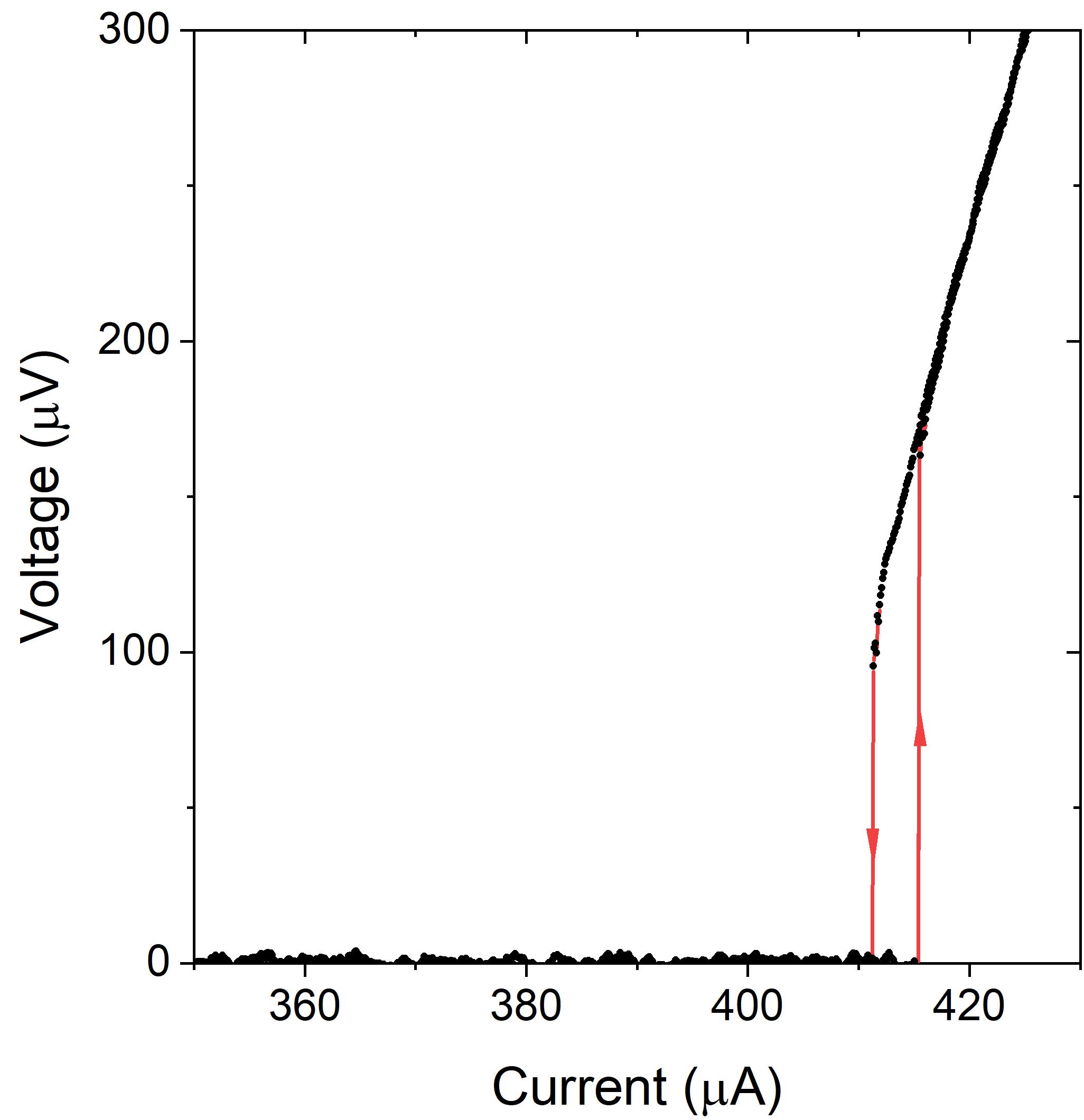 |
| --- |
| **Figure S5\|** *IV* curve of 76-nm-wide, 530-nm-long, and 9-u.c.-thick Au/YBCO nanowire at *T* = 4.2 K. |

| 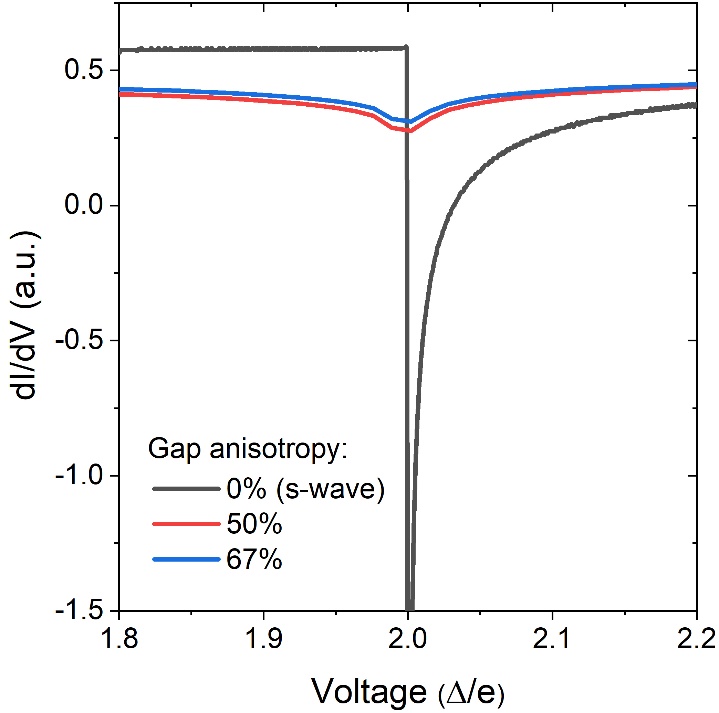 |
| --- |
| **Figure S6\|** Numerically simulated differential conductivity of the voltage-biased SNS junction for different values of the anisotropy of the order parameter. Simulation parameters: reduced temperature *t* = 0.05*T_c_*, reduced junction length *d* = 3*ξ*, and reduced quasiparticle mean-free path *l* = 4.5*ξ*, where *T_c_* is the critical temperature and *ξ* – is the coherence length*.* |

| 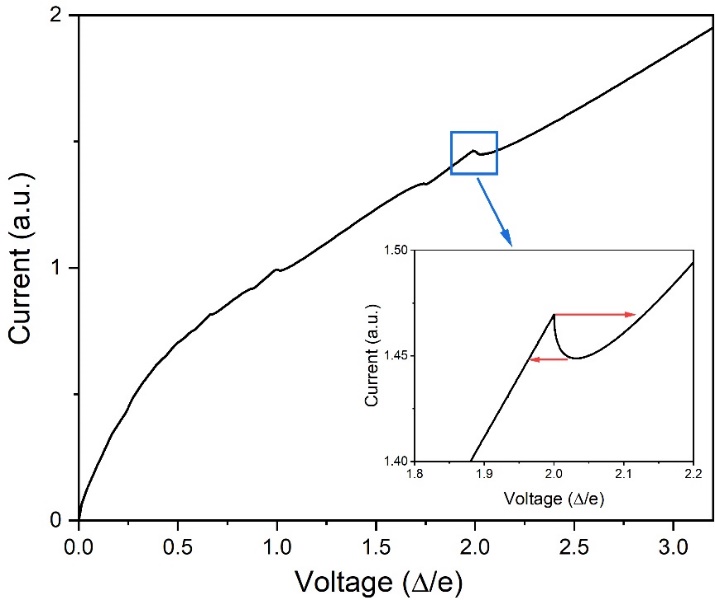 |
| --- |
| **Figure S7\|** Numerically simulated current-voltage characteristic of the voltage-biased SNS junction. Inset. Zoomed current-voltage characteristic. Red arrows show the voltage switching in the current-bias regime. |

| 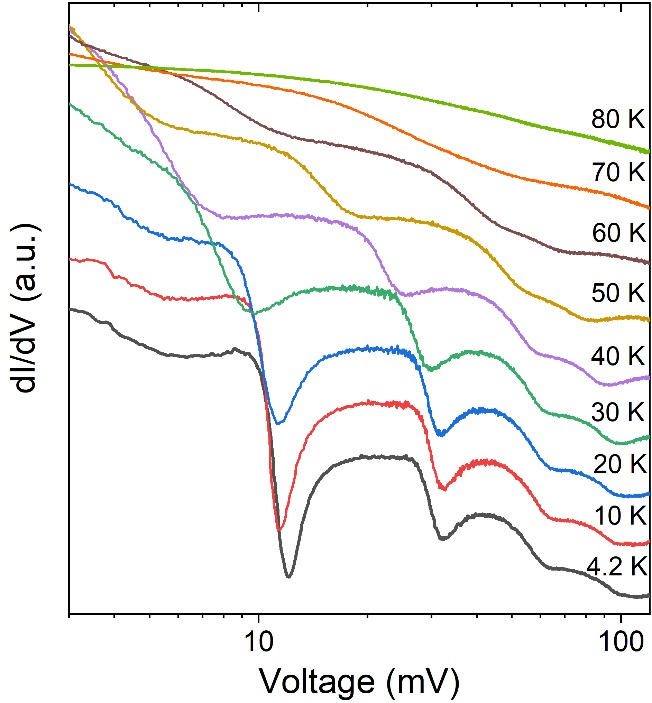 | 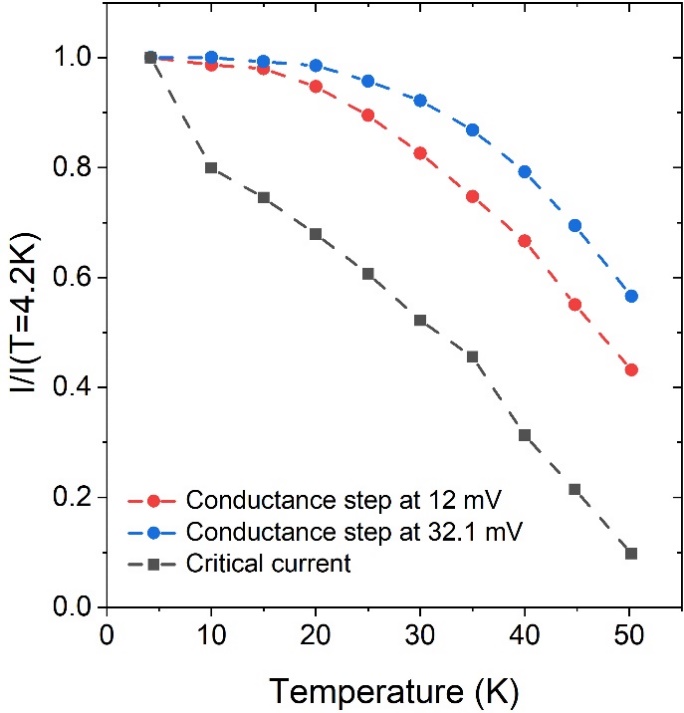 |
| --- | --- |
| **Figure S8\|** Differential conductance of the ultra-narrow 6-u.c.-thick YBCO nanoconstriction capped by the amorphous YBCO layer at various temperatures. Curves are shifted along the Y-axis for convenience of their representation. | **Figure S9\|** Temperature dependences of critical current and current position of the conductance steps at 12 mV and 32.2 mV of the ultra-narrow 6-u.c.-thick YBCO nanoconstriction capped by the amorphous YBCO. |

| 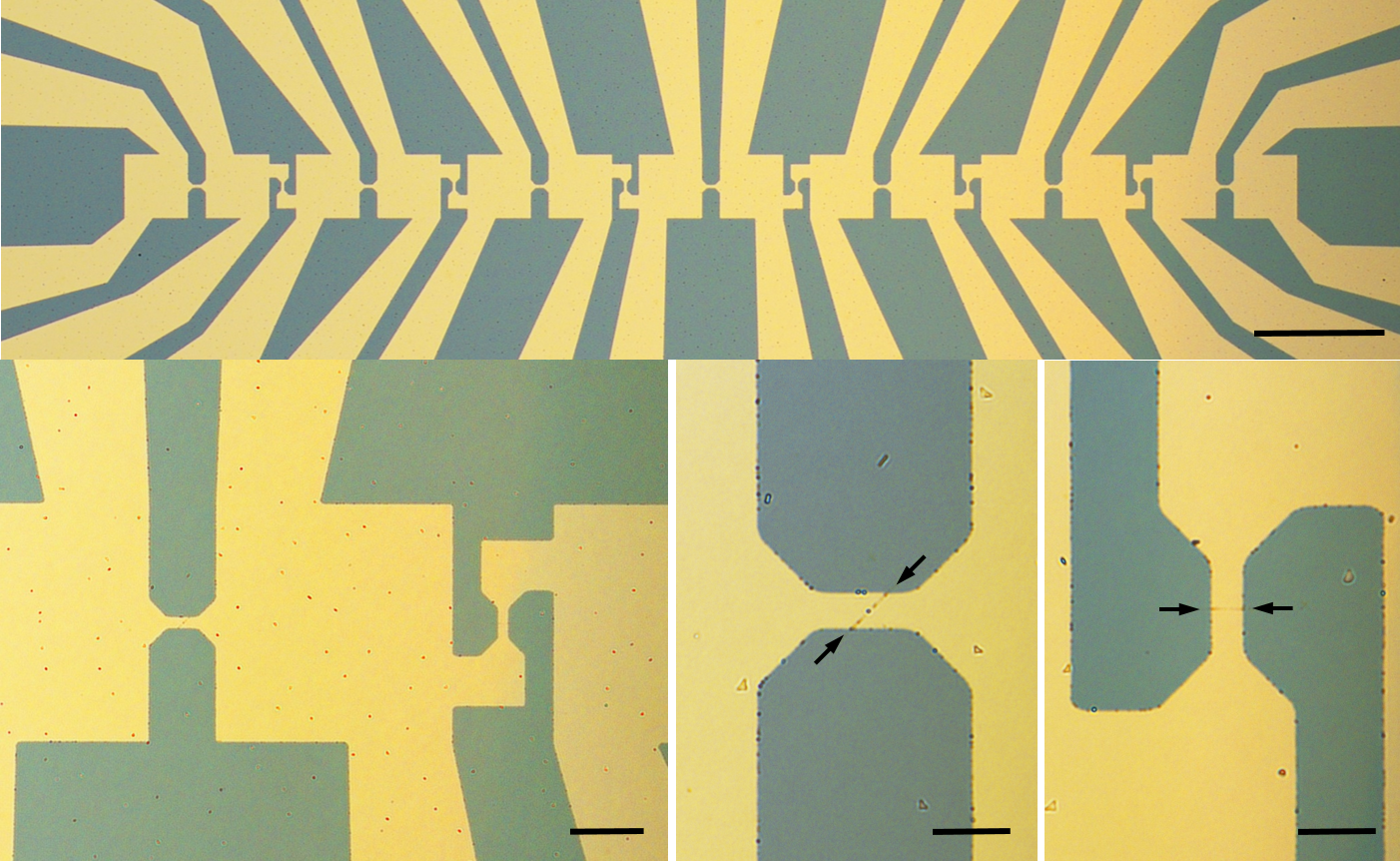 |
| --- |
| **Figure S10\| Optical micrographs.** a. Sample layout. Scale bar is 200 μm. b. Microbridges aligned along different substrate edges. Scale bar is 30 μm. c. Zoomed image of the microbridge with “horizontal” orientation. Scale bar is 10 μm. d. Zoomed image of the microbridge with “vertical” orientation. Scale bar is 10 μm. The nanowire orientation is shown by black arrows. |
